# Nasal reconstruction surgery after continuous positive airway pressure delivered by prongs: A case report

**DOI:** 10.1016/j.amsu.2021.102881

**Published:** 2021-09-24

**Authors:** Carolus Aldo Windura, Fonny Josh, Tomie H. Soekamto, Dhevie Gianfranco Lumalessil

**Affiliations:** a Division of Plastic and Reconstructive Surgery, Department of Surgery, Faculty of Medicine, Hasanuddin University, Makassar, Indonesia; b Division of Plastic and Reconstructive Surgery, Department of Surgery, Dr. Wahidin Sudirohusodo Hospital, Makassar, Indonesia

**Keywords:** Case report, Nasal deformity, Nasal reconstruction, nCPAP, nCPAP, nasal prongs continuous positive airway pressure, NICU, neonatal intensive care unit, FTSG, full thickness skin grafts

## Abstract

**Introduction and importance:**

Deformities resulting from nasal continuous positive airway pressure delivered using prongs can cause functional and aesthetic issues for patients. Resultant severe tissue damage to the nasal structures often requires surgical intervention and techniques continue to evolve.

**Case presentation:**

This case report describes a 6-year-old male presenting with a full-thickness columella defect; contracture causing deformities involving the nasal tip, ala nasi, and left nasal cavity wall; missing left lateral-medial cruris cartilage; and partially missing right medial cruris cartilage. The abnormalities initially appeared when the patient was 7 days old after receiving treatment by nasal continuous positive airway pressure for 7 days. A one-stage procedure was performed as follows: left ala nasi reconstruction with skin excision followed by an ear cartilage graft; a nasal cartilage shield graft to form the nasal tip; reconstruction of the columella with a cartilage graft combined with bilateral soft tissue flaps taken from the nasal floor and mucosa vestibulum; and a full-thickness skin graft to cover the secondary defect resulting from the flaps. At 1-month post-surgery, satisfactory results were reported.

**Clinical discussion and conclusion:**

Our approach combining two flaps taken from the nasal floor and the inner layer of the ala nasi, a cartilage graft from the ear, and a full-thickness skin graft delivered a one-stage surgical technique that yielded satisfactory results without deformities of the donor site. However, the surgical technique should be individualized to patients. This case report adds to the literature and offers surgeons an alternative approach for managing nasal deformities.

## Highlights

• The limited availability of small size nCPAP in inland hospitals have caused the increased number of nasal deformity.
• Combination of nasal floor flaps, shield and ear cartilage graft yielded satisfactory result for nasal reconstruction.
• The rarity of the case, and the combination of each techniques used, made the case interesting.
• This technique does not rule out the possibility of secondary corrections and should be tailored individually for each case.

## 1 Introduction

The nose is a complex facial structure that has major functional and aesthetic roles. Nasal deformities can result in issues that might lead to future social and psychological disorders. Those that are not congenitally inherited, are commonly caused by iatrogenic actions; this include prolonged use of nasal continuous positive airway pressure delivered by prongs [1].

Related deformities can be caused by using a cannula that is too large or applying excessive pressure that results in local ischemia. In addition, excessive movement due to poor immobilization techniques can cause skin erosion, which may progress to ulceration resulting in loss of the surrounding tissue. Fischer reported that the use of nCPAP caused nasal trauma in 42.5% of treated patients, with 0.7% of cases having tissue necrosis with full-thickness skin loss [[1], [2], [3]].

The use of a combination of skin grafts and flap techniques has largely been determined by the structure of the missing tissue and the severity of the deformities. To our knowledge, there has been no reported algorithm to specifically regulate columella reconstruction. The appropriate procedure therefore needs to be individualized to the patient in every case [4,5].

The primary aim of the reconstructive surgery reported here was to preserve the aesthetic features of the face [1,4]. Such case reports remain rare and research into surgical techniques that can deliver satisfactory results are ongoing. Our patient presented with a nasal deformity resulting from nCPAP insertion 6 years previously, and he underwent columella and nasal septum reconstruction followed by correction of the ala nasi. This work adhered to the 2020 Surgical Case Report guidelines [6].

## 2 Presentation of case

**Fig. 1:**
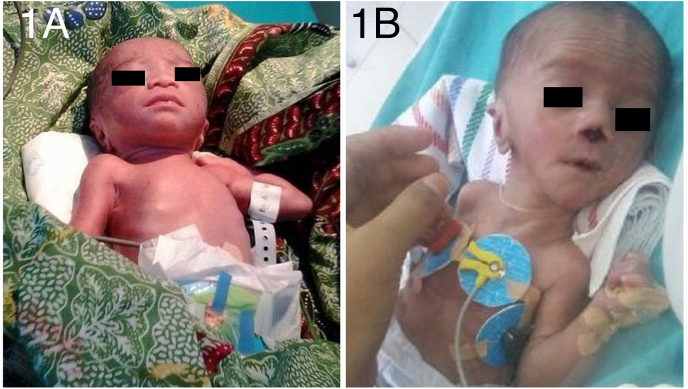
(A) Photograph showing normal nose shape of the patient at birth. (B) Photograph showing a reddish wound, black necrotic tissue, and nasal deformity after the patient had been treated using nCPAP for 7 days.

The 6-year-old male patient presented with deformities of the nose. He had been born with typical nasal morphology (Fig. 1A), problems had been noted after 7 days of treatment by nCPAP in the neonatal intensive care unit. On the 7th day of hospitalization, a reddish wound had appeared on the patient's left columella and ala nasi, which was accompanied by tissue ulceration that was partially black in color (Fig. 1B). By the time the patient was 2 months old, the redness and ulceration had healed with minimal scarring; however, the necrotic tissue resulted in a persistent nasal deformity thereafter.

The patient did not have any systemic disorders. There was no recorded history of treatment. No history of smoking, drug consumption, or other gestational issues. The mother had suffered from severe preeclampsia during pregnancy. Owing to the severity of the preeclampsia, the patient had been delivered at 28 weeks of pregnancy by caesarean section, with a birth weight of 1200 g, and had been admitted to the NICU for 2 months.

**Fig. 2:**
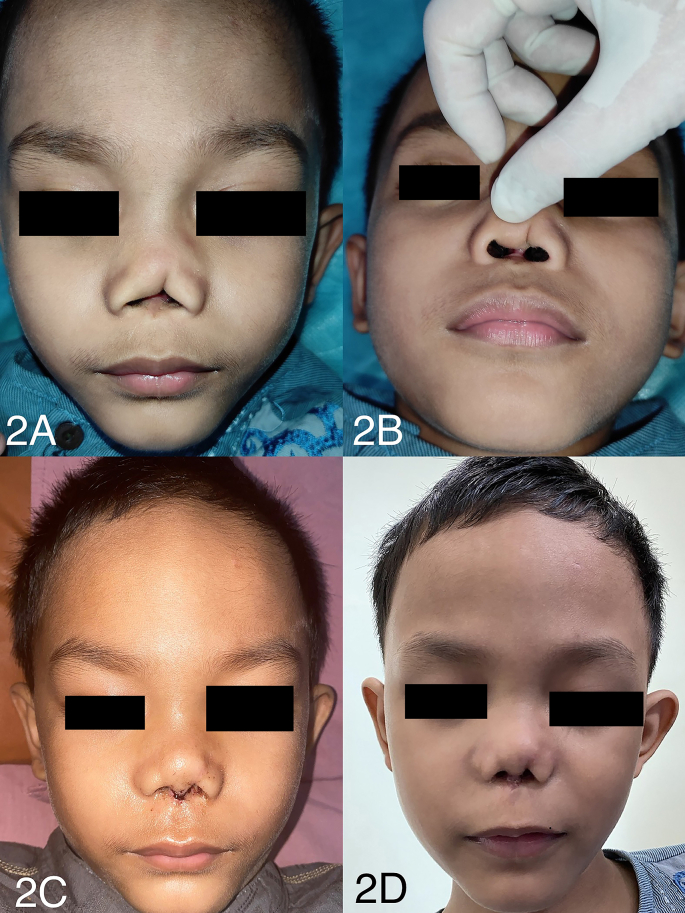
(A) Photograph of the patient before surgery — frontal view. (B) Photograph of the patient before surgery — worm position. (C) Photograph of the patient 5 days after surgery. (D) Photograph of the patient 1 month after surgery.

A physical examination revealed a full-thickness columella of 10 mm × 0.8 mm (the size of the missing nasal septum was 0.8 mm). We observed contracture-like deformity of the nasal tip, ala nasi, and left cavum nasi wall; this had resulted in irregularities of the nasal tip shape as well as asymmetry of the right and left ala nasi (Fig. 2A and B). The patient's left lateral cruris cartilage was found to be missing upon palpation. A portion of the left medial cruris cartilage along with the mucocutaneous tissue that forms the columella were absent.

We treated this case with a one-stage procedure. In brief, the skin of the nasal tip was incised and the mucosa and skin were separated, which revealed that the medial and lateral cruris of the left cartilage were absent. Some of the right medial cruris cartilage and all of the right lateral cruris cartilage were present. A cartilage graft was provided by a donor from the left ear concha using a retro-auricular approach, and comprised the left lateral cruris cartilage, medial ala nasi, part of the right medial cruris cartilage, and the columella. The cartilage graft was sutured using 5.0 nylon thread. The shield ear cartilage graft on the dome was fixated to the medial cruris cartilage graft below (Illustration 1A); the contracture on the left ala nasi was then released and the skin excess was excised. The wound was sutured using non-absorbable 6.0 thread (Illustration 1B).

**Illustration 1:**
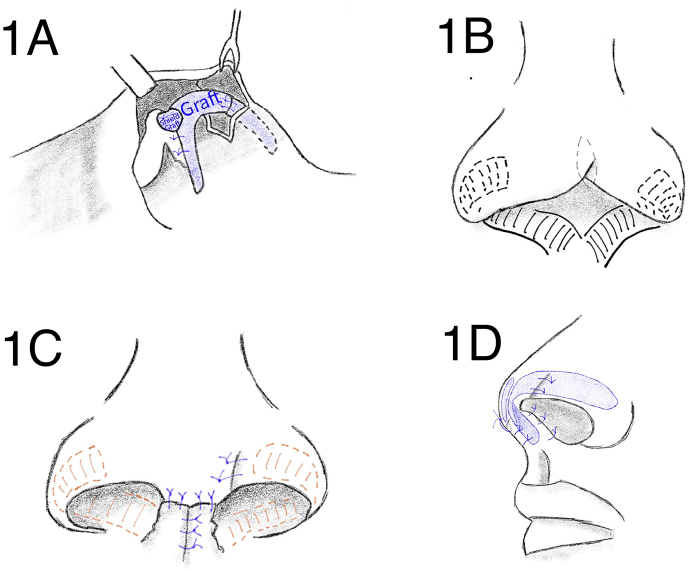
(A) Cartilage graft to form the lateral and medial crus of the left lower lateral cartilage. This graft was fixated to the right lower lateral cartilage. (B) Shaded area shows flap design which will form the columella. (C) Shaded area is a defect due to the donor site of the flap, which then grafted with full thickness skin graft. (D) End result of the procedures from lateral view.

Columella reconstruction was performed using bilateral skin soft tissue from the nasal floor that extended to the inner layers of the ala nasi. The pedicle of the flap was located directly above the philtrum, which was predicted to be the appropriate position of the columella. An incision was made proportional to the flap design. The distal end of the flap with the inner layer of the nasal tip was then sutured to form the outer layer of the nasal septum. A cartilage graft was placed between the flaps to shape the columella (Illustrations 1C and 1D). A full-thickness skin graft from behind the ear was performed to close the secondary bilateral flap defect.

No complications occurred. At the 5th-day post-surgery follow-up, the flap was viable, and graft evaluation was performed with a result of fully viable. The sutures were removed on day 5 (Fig. 2C) except for the sutures at the distal columella flap, which were retained until day 7 after the surgery The patient's parents were satisfied with the results of the surgery (Fig. 2D). All procedures were conducted by our team of plastic and reconstruction surgeons in hospital setting.

## 3 Discussion

Tissue damage due to nCPAP has been reported ever since the 1950s and continues to be described to date. Surgical techniques have also evolved and various methods are currently available as a result of the continuing search for a comprehensive surgical technique. Our current report is no exception [7,8].

An optimal nasal reconstructive surgery approach should preserve the aesthetic subunits of the nose; cause minimal scarring; provide a suitable match between the donor and the recipient in terms of the texture, color, and profile of the tissue; create minimal damage to the donor site; and carry a low risk of complications. Deciding on the correct surgical technique requires careful planning and should take all these factors into consideration [1,9].

The columella forms a line that defines the nasolabial angle point and the inferomedial border of the nose [1]. The patient had lost his entire columella and part of the anterior nasal septum, so the nasolabial angle was not formed. The columella and nasal tip consist of three layers: an inner layer, which comprises skin mucosa; a middle layer, which comprises cartilage; and an outer layer, which comprises skin [[10], [11], [12]]. These unique structures must be restored to reconstruct the subunits. In this case, we used a bilateral skin-soft tissue flap surgical technique taken from the cutaneous tissue of the nasal floor, which extended to the right and left inner layers of the ala nasi (Illustration 1B). This technique, initially described by Vecchione [13].

Flap modification was performed by transposition to form the basal layer of the left and right nasal septum, including the columella. Furthermore, by placing the cartilage graft between the two flaps, the columella was thickened and had a more natural appearance (Illustrations 1C and 1D). The flap on the nasal vestibule mucosa in this case was longer and wider than the conventional technique, which adjusted for the extent of the missing tissue.

In our case, a FTSG was performed to close the secondary defect. This procedure was intended to limit the wound-contraction process during healing and to preserve the shape of the ala nasi, thereby minimizing scarring and reducing the future risk of hypertrophic scars or keloid formation [13,14].

A graft was inserted to replace the necrotic medial and lateral cruris cartilage. In addition, a shield graft was used to sharpen the three-dimensional projection of the nasal tip, which had previously been lost (Illustration 1A). Cartilage and FTSG skin grafts were harvested from the donor's ear via a retro-auricular approach to produce a hidden scar leaving minimal deformity [15,16].

Symmetry of the left–right ala nasi and the patient's nasal tip was achieved by releasing the contracture scar; as this procedure resulted in excess skin, a cut-as-you-go excision was performed to ensure that the nasal tip appeared symmetrical. The excision line was positioned according to the location of the soft-triangle facet, with the intention that any resultant scarring would fall within the facet cavity and thus be imperceptible [17,18].

Evaluations made 5 days and 1-month after the surgery met the following benchmarks: right and left ala nasi symmetry; nasal protrusion with a diamond shaped tip; and a columella forming a nasolabial angle of approximately 120° (Fig. 2C) [[17], [18], [19]].

Long-term evaluation remains necessary and do not rule out the possibility of secondary corrections due to the ongoing processes of bone and associated soft-tissue growth [20]. In addition, the surgical technique used should be tailored individually.

## 4 Conclusion

This case report described a nasal deformity attributed to the use of nCPAP. The combination of two flaps, shield graft, alongside an ear cartilage graft and FTSG, delivered a one-stage surgical technique that yielded satisfactory results without deformity of the donor site.

## Ethical approval

There is no such ethical approval needed for this type of publication in our institution. However, the parents have given their approval for this publication.

## Funding

This research did not receive any specific grant from funding agencies in the public, commercial, or not-for-profit sectors.

## Consent

Patient's parents have given consent for publication of this case report and accompanying images.

## Author contribution

Carolus Aldo Windura: Editing, writing, data collection.

Fonny Josh: Study concept, writing original draft preparation, review, surgical therapy.

Tomie Hermawan Soekamto: Study concept, surgical therapy.

Dhevie Gianfranco Lumalessil: Editing, writing.

## Guarantor

Fonny Josh.

## Provenance and peer review

Not commissioned, externally peer-reviewed.

## Declaration of competing interest

The author(s) declare that there are no competing interests.
